# Placental blood flow sensing and regulation in fetal growth restriction

**DOI:** 10.1016/j.placenta.2021.01.007

**Published:** 2021-09-15

**Authors:** L.C. Morley, M. Debant, J.J. Walker, D.J. Beech, N.A.B. Simpson

**Affiliations:** a Leeds Institute of Cardiovascular and Metabolic Medicine, University of Leeds, LS2 9DA, UK; b Division of Women's and Children's Health, School of Medicine, University of Leeds, LS2 9NS, UK

**Keywords:** Placenta, Shear stress, Flow, Mechanosensing, Piezo1, Endothelial cell

## Abstract

The mechanical force of blood flow is a fundamental determinant of vascular homeostasis. This frictional stimulation of cells, fluid shear stress (FSS), is increasingly recognised as being essential to placental development and function. Here, we focus on the role of FSS in regulating fetoplacental circulatory flow, both in normal pregnancy and that affected by fetal growth restriction (FGR).

The fetus is reliant on placental perfusion to meet its circulatory and metabolic demands. Failure of normal vascular adaptation and the mechanisms enabling responsive interaction between fetoplacental and maternal circulations can result in FGR. FSS generates vasodilatation at least partly through the release of endothelial nitric oxide, a process thought to be vital for adequate blood flow. Where FGR is caused by placental dysfunction, placental vascular anatomy is altered, alongside endothelial dysfunction and hypoxia, each impacting upon the complex balance of FSS forces.

Identifying specific mechanical sensors and the mechanisms governing how FSS force is converted into biochemical signals is a fast-paced area of research. Here, we raise awareness of Piezo1 proteins, recently discovered to be FSS-sensitive in fetoplacental endothelium, and with emerging roles in NO generation, vascular tone and angiogenesis. We discuss the emerging concept that activating mechanosensors such as Piezo1 ultimately results in the orchestrated processes of placental vascular adaptation. Piecing together the mechanisms governing endothelial responses to FSS in placental insufficiency is an important step towards developing new treatments for FGR.

## Graphical abstract

**Figure:**
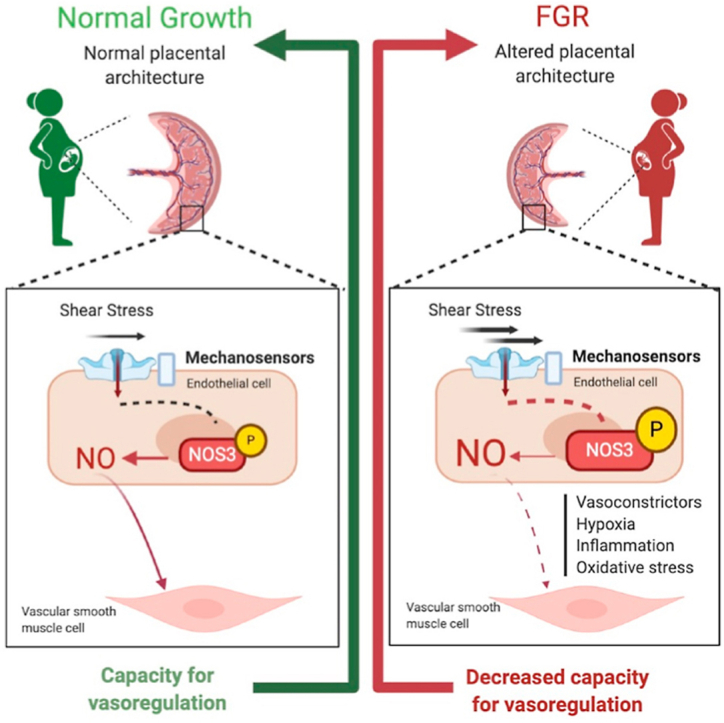

## Abbreviations

cGMP: Cyclic guanosine monophosphate
EC: Endothelial cell
eNOS: Endothelial nitric oxide synthase
FGR: Fetal growth restriction
FMV: Flow mediated vasodilatation
FpEC: Fetoplacental endothelial cell
FSS: Fluidic shear stress
GTP: Guanosine triphosphate
hCAT-1: High affinity cationic amino acid transporter 1
l-NAME: Nω-Nitro-l-arginine methyl ester hydrochloride
L-NMMA: Nω-Monomethyl-l-arginine acetate
NO: Nitric oxide
PECAM-1: Platelet endothelial cell adhesion molecule 1
PlGF: Placental growth factor
PKG: Protein Kinase G
siRNA: Short interfering RNA
SNP: Sodium nitroprusside
VEGFR: Vascular endothelial growth factor receptor

## 1 Introduction

The placenta is the interface between mother and fetus. It undergoes constant vascular change and differentiation in order to oversee and maintain effective interplay between the uteroplacental and fetoplacental circulations to ensure the health of the baby. The fetus is totally reliant on placental perfusion for effective oxygenation and nutrient supply. When blood flow is compromised, the circulatory and metabolic demands of the fetus may not be met, and fetal growth restriction (FGR) can result. FGR has been defined as where a fetus ‘does not meet its biological growth potential as a consequence of placental dysfunction’ [1]. Affected pregnancies are associated with perinatal morbidity and mortality [2]. Long term impacts include increased risks of obesity, metabolic and cardiovascular disease into adulthood [3].

The aetiology of FGR is complex and multifactorial, even for the majority of cases that are of placental origin. Characteristic features include fetoplacental hypoperfusion, hypoxia and high vascular resistance, with the degree of abnormality proportionate to placental compromise [2]. Microscopic and stereologic features commonly include structural vascular abnormalities, such as villous immaturity and infarction, and decreased villous density [4,5]. Critical to developing effective therapies for fetal growth restriction (FGR) is a fundamental understanding of the molecular mechanisms responsible for fetoplacental vasoregulation and how they may be manipulated. Due to the lack of autonomic innervation, fetoplacental vasculature is locally regulated by the mechanical force of blood flow, fluid shear stress (FSS) and the variable release of paracrine and vasoactive mediators [6]. Recent advances have shed new light on the molecular controllers of haemodynamic force sensing on the fetoplacental endothelium, and their links to downstream pathways leading to vascular adaptation. This review focuses on the regulation of fetoplacental circulatory flow in normal pregnancy and FGR, and the implications for therapeutic intervention.

## 2 Haemodynamic force in fetoplacental blood vessels

From as early as the embryonic heart starts beating, FSS is a critical determinant of vasculo- and angiogenesis, triggering endothelial cells (ECs) to develop a vascular network [7]. Such is the degree of vascular expansion in the human placenta, that by term there is a ten-fold increase in the villous volume occupied by vasculature [8]. The network of vessels from each umbilical artery via the chorionic vessels extends into 60–100 individual villous trees [9]. Terminal arborisation creates a capillary network enabling maximal gas exchange, nutrient and waste transfer [9]. The establishment and remodelling of this vascular network results in a high flow, low resistance circuit, enabling effective perfusion in the absence of hypertension [10]. Structural change on its own, however, does not explain how the placenta autoregulates blood flow to meet localised oxygen demands, enabling minute-to-minute fluctuations in the perfusion of its multiple villous trees.

As pregnancy progresses, the fetoplacental endothelium is constantly exposed to haemodynamic force. During each cardiac cycle, varying blood flow results in shearing forces on the ECs [10]. This FSS is dependent on vessel calibre, flow rate and blood viscosity [11]. When exposed to FSS, fetoplacental endothelial cells (FpECs) exhibit morphological changes, elongating and re-orientating to the direction of flow [12].

Efforts to accurately investigate FSS in fetoplacental microvasculature have been complicated by its inaccessibility to *in vivo* high resolution imaging [9], additional to the challenges caused by flow pulsatility and complexity of villous architecture [9,13]. Variations in umbilical cord insertion point and vessel branching pattern, for example, will produce differing intraluminal forces [9]. Correspondingly, a computational fluid dynamics model of the rat placenta found heterogenous FSS throughout the vascular network, with gradients at vessel bifurcations [14]. High fidelity *in silico* models of human fetoplacental haemodynamics will therefore be a valuable tool for providing metrics that can be correlated with *in vivo* fetoplacental assessment [15].

## 3 Fluid shear stress induces production of EC-derived vasoactive mediators

### 3.1 Nitric oxide (NO)

**Fig. 1:**
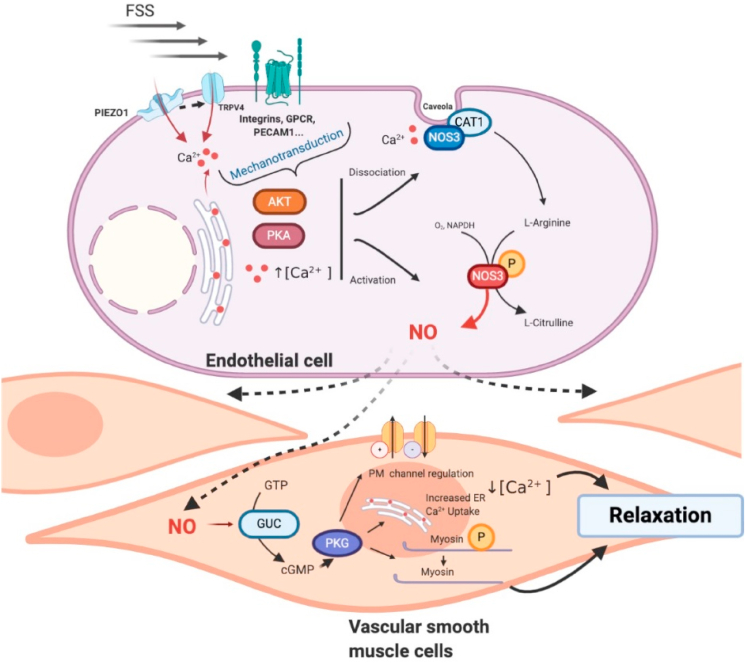
**Schematic illustrating possible mechanisms by which FSS-induced mechanosensor activation results in vasodilatation through the production of NO.** Example mechanosensory components of the endothelial cell presented in this figure include Piezo1 and TRPV4 ion channels, G-protein coupled receptors (GPCR), integrin receptors, and cell-cell junction proteins such as platelet adhesion cell molecule 1 (PECAM-1) [25,41]. Other abbreviations: NO nitric oxide, P phosphorylation, NOS3 endothelial NO synthase. Created with BioRender.com.

It has long been established that increasing flow reduces placental vascular resistance [16,17]. FSS is the most powerful physiological stimulator of endothelial nitric oxide synthase (eNOS/NOS3), which when activated leads to generation of NO (Fig. 1) [10]. This constitutively-produced mediator is known to be a potent vasodilator within placental vasculature [17].

The activity of eNOS is dependent on Ca^2+^, both from rapid release from endoplasmic reticulum storage, and sustained influx across the plasma membrane [18]. As such, placental Ca^2+^ transport is a key determinant of NO-driven vasodilatation [19]. When quiescent, eNOS is bound to caveolae, co-localised with amino acid transporter proteins such as CAT1 (Fig. 1). Increasing intracellular Ca^2+^ results in eNOS being liberated from these caveolae [18]. Further association of eNOS with kinases such as AKT (Protein kinase B) and protein kinase A induces phosphorylation and thereby activation of eNOS at serine residue 1177 [18].

The conversion of l-arginine, NADPH and oxygen to l-citrulline, NADP^+^ and H^+^ is catalysed by eNOS, with NO formed as a by-product (Fig. 1). Once generated, NO diffuses and binds to guanylate cyclase on smooth muscle cells, catalysing the dephosphorylation of GTP to produce cyclic GMP [10]. Sustained Ca^2+^ influx into the EC is therefore required to maintain eNOS in the cytosol [18]. Multiple downstream pathways lead to vasodilatation, including the activation of protein kinase G (PKG), and subsequently, myosin phosphatase (Fig. 1). Conversely, phosphodiesterases remove cGMP by degrading is phosphodiester bond, suppressing the NO signalling cascade.

In placental perfusion models, flow induces NO release [17]. Correspondingly, pre-treating chorionic arteries with the eNOS inhibitors l-NAME or L-NMMA (Nω-Nitro-l-arginine methyl ester hydrochloride, Nω-Monomethyl-l-arginine acetyl salt) significantly reduces flow-mediated vasodilatation [10]. Furthermore, this can be reversed by adding the eNOS substrate, l-arginine [10].

### 3.2 Vascular endothelial growth factor (VEGF)

The interplay between VEGF activity and NO bioavailability in fetoplacental vasculature is incompletely understood. In animal models, l-arginine supplementation increased VEGF expression and subsequent systemic angiogenesis, suggesting that vascular remodelling via VEGF involves the NO pathway [11]. In HUVECs, VEGF treatment produced a concentration-dependent rise in cGMP that was inhibited by l-NAME [20]. Correspondingly, VEGF incubation increased eNOS protein and angiogenesis (tube formation), which were both inhibited by l-NAME. Furthermore, inhibiting tyrosine kinases and applying Ca^2+^ chelators attenuated VEGF-induced NO release [20].

More recent HUVEC data suggest that VEGF receptors are part of a FSS-sensing complex with vascular endothelial cadherin (VE-cadherin) and platelet endothelial cell adhesion molecule (PECAM-1) [21,22]. This results in VEGFR-2 phosphorylation, activating AKT/Protein kinase B and the signalling cascade which produces NO [23].

### 3.3 Adenosine triphosphate (ATP)

HUVEC data demonstrate that endothelial ATP release is also flow-stimulated [23]. Correspondingly, apyrase (which degrades ATP) inhibits the FSS-induced Ca^2+^ influx. ATP binds to the P2Y_2_ receptor on ECs, which is coupled to G_q_ and G_11_ proteins. Endothelial P2Y_2_/G_q_/G_11_ subsequently activates the VE-cadherin, PECAM-1 and VEGFR-2 triad, leading to AKT/Protein kinase B phosphorylation and NO release [23]. Although evident that flow-induced ATP release is a mechanism upstream of NO vasodilatation, little is known about its role in fetoplacental endothelium.

## 4 Mechanisms of fetoplacental blood flow sensing

Given the strong association of FSS with downstream production of vasoactive mediators, understanding how the haemodynamic environment is sensed by the fetoplacental endothelium is essential. A growing body of literature is dedicated to identifying FSS sensors, including proteins, receptors, transmembrane channels and components of the cell architecture (Fig. 1), a selection of which are reviewed here in brief.

### 4.1 EC structures

The surface of ECs has been described as a ‘flexible signalling hub’ [24]. Protein filaments of the cytoskeleton such as vimentin may be deformed by flow, impacting on multiple cellular components, such as integrins, adhesion proteins, and the extracellular matrix, where FSS is transduced [25]. Force may also be transmitted to the cytoskeleton via glycocalyx moieties on the EC membrane [25].

Caveolae membrane invaginations are abundantly expressed on the surface of ECs, but not in the trophoblast [11,26]. *CAV-1* gene expression has been found in both HUVECs and microvascular fetoplacental ECs (FpECs) [11]. In systemic ECs, exposure to FSS increased both the amount of caveolae, and *cav-1* expression. In HUVECs and ovine FpECs, *CAV-1/cav-1* knockdown reduced NO production and VEGF-induced tube formation. The link between this and the NO pathway may lie in eNOS co-localising with CAT1 in the caveolae [11,27,28].

### 4.2 Ion channels

The endothelium expresses an array of ion channels and identifying those that sense FSS in the placenta is an emerging area. Subtypes of K^+^ channels have been demonstrated in chorionic plate vessels and villous homogenate, including voltage-gated (K_v_), large conductance Ca^2+^ (BK_Ca_), and ATP-sensitive (K_ATP_) channels, which are oxygen-sensitive [29]. In HUVECs, insulin-induced l-arginine transport and membrane hyperpolarisation were attenuated by a K_ATP_ blocker [11]. However, a role for K^+^ channels in placental mechanosensing remains to be determined.

Piezo1 mechanosensitive cation channels are critical to vascular development and survival in mouse models with a disrupted endogenous *Piezo1* gene [30,31]. Since its discovery in 2010, Piezo1 has risen to prominence as a key channel in FSS sensing [32]. Their activation is thought to be directly modulated by membrane tension, leading to Ca^2+^ influx into the EC [33]. Our group has reported Piezo1 gene and protein expression in FpECs and HUVECs [12,30]. Piezo1 depletion using siRNA reduced eNOS, and abolished VEGF-evoked eNOS phosphorylation [30]. The mechanism by which FSS-activated Piezo1 leads to NO release is under investigation. It has been suggested that Piezo1 mediates flow-induced ATP release [21]. This activates the P2Y_2_ receptor and G proteins, leading to NO production. The potential role of Piezo1 in NO-driven fetoplacental vasodilatation is therefore an exciting area of research.

### 4.3 Mechanosensory complexes

Multiple FSS sensors may be co-dependent, forming mechanosensory complexes such as the PECAM-1/VE-cadherin/VEGFR-2 triad [25,34]. PECAM-1 junctional proteins are thought to transmit FSS to the VE-cadherin receptor, which functions as an adaptor, and recruits VEGFR-2 [34]. This triggers kinase phosphorylation, leading to NO production. Three dimensional electron microscopy images of human placental villi have recently demonstrated inter-endothelial protrusions originating at the endothelial junction and projecting deeply into adjacent ECs [35]. Determining whether these *trans*-EC connections facilitate mechanosensing is an exciting prospect.

Intermediate filaments, such as vimentin alter the tension on PECAM-1 when disturbed by flow [36]. Both PECAM-1 and vimentin are readily detected in FpECs, although the presence of a mechanosensory complex involving these proteins in the placenta remains unknown. Furthermore, immunohistochemical staining of placental tissue from women with severe pre-eclampsia has shown increased intravillous vimentin, with expression clustered around sites of chorionic vessel damage [36]. Whether this augmentation of vimentin is a reaction to pathological FSS, or if ultrastructural changes caused by the upregulation of cytoskeletal proteins affect the responsiveness to flow, remains to be determined.

## 5 Vascular maladaptation and compensation in FGR

### 5.1 Impaired flow mediated vasodilatation in FGR

*In vivo* assessment of placental function in FGR relies on umbilical artery Doppler ultrasonography, where altered flow velocity waveforms and increased pulsatility are indicative of increased downstream vascular resistance. In the FGR placenta, structural abnormalities including altered villous branching, in combination with vasoconstriction, may raise the transmural pressure [37]. A computational model of placental microvasculature has estimated FSS to be increased in FGR (0.05 Pa in the normal placenta versus 0.2 Pa in severe FGR), representative of a five-fold elevation in total placental vascular resistance [38].

Increased FSS in FGR is supported by perfusion model data. In placental samples from normal pregnancies, the lowest measures of *in vivo* resistance on umbilical artery Doppler velocimetry correlated with maximal flow through the fetoplacental circuit [16]. Increasing the flow reduced fetal-side hydrostatic pressure, demonstrative of flow mediated vasodilatation [16]. In FGR samples, baseline vascular resistance was markedly elevated, and furthermore, flow mediated vasodilatation was substantially reduced or absent [16].

Myography of chorionic arteries showed vasoconstriction to a thromboxane mimetic (U46619), and relaxation in response to the NO donor SNP (sodium nitroprusside) [39]. Vessels from FGR placentas showed enhanced vasoreactivity, displaying both increased contraction in response to U46619, and dilatation with SNP. In the perfusion model, inhibiting eNOS with l-NAME in FGR placentas caused an increase in vascular resistance which far exceeded the response in normal tissue [16]. As such, Jones et al. (2015) argue that ‘vessels from dysfunctioning placentae have the capacity to vasodilate over-and-above those from a healthy pregnancy’ [16]. Chorionic artery ECs produced nitrite, and thus NO, proportionate to the level of FSS. Nitrite concentrations were significantly greater in FGR cells exposed to high FSS [16]. Increased eNOS protein expression in FGR has also been demonstrated in numerous studies [8,16,40].

Taken together, findings from whole vessels and ECs imply that despite impaired flow mediated vasodilatation, FGR placental vasculature shows increased NO, eNOS and responsiveness to NO. These enhanced components of the NO system are suggestive of an adaptive physiological mechanism for overcoming deficiencies in the fetoplacental circulation [40]. However, when endothelial dysfunction is severe enough to prevent this response to increased FSS, flow-induced NO compensation may be insufficient and vascular dysregulation may still progress [16,41]. Added to this is the knowledge that NO at high concentrations combines with superoxide to form peroxynitrite, which leads to the production of nitrotyrosine, known to cause nitrative stress and inflammation [42].

Also contributing to abnormal vasodilatation in FGR are increased vasoconstrictors, such as endothelin-1, and lower prostanoid synthesis related to altered endothelial expression of oestrogen receptor-β [43]. This highlights the complexity of factors influencing both flow-mediated, and flow-independent, vasoregulation.

### 5.2 Interplay between hypoxia and endothelial dysfunction in normal and FGR pregnancy

Compensatory flow-induced NO in FGR is dependent upon the production and response to vasoactive mediators by the endothelium. In a study of HUVECs from FGR placentas, gene expression for the CAT proteins involved in eNOS activation was impaired, alongside reduced l-arginine transport and l-citrulline production [27]. The activity of arginase-2, the enzyme competitor of eNOS was increased in these cells, thus influencing the bioavailability of l-arginine for NO production. A comparable increase in arginase-2 was also seen after exposing HUVECs to hypoxia (13.5 mmHg versus 33.9 mmHg normoxia control), suggesting that lower oxygen tensions in the FGR placenta may upregulate arginase-2, reducing the ratio of phosphorylated eNOS to arginase-2 [37].

The relationship between hypoxia and FSS sensing remains to be determined. Perfusion data suggest that hypoxia increases vascular resistance, an effect which could be enhanced or inhibited by modulators of the oxygen-sensitive K^+^ channels previously described [29,44]. In the systemic circulation, altering K^+^ channel activity is associated with vasoconstrictive hypertension. K^+^ channels are regulated by reactive oxygen species (ROS), known to be elevated in the FGR placenta [44]. In addition to FSS, low oxygen tension also drives ATP production [45]. Given that purinergic signalling is one mechanism of endothelial NO production, this could indicate a compensatory drive towards vasodilatation in the placenta.

## 6 Modulating haemodynamic regulation for treating FGR

Enhancement of physiological FSS through exercise has shown beneficial effects in the systemic circulation, whereby flow-induced increased NO is associated with improved cardiovascular disease outcomes [11]. One study of maternal exercise on placental NO, found higher eNOS expression in whole villous homogenate, along with reduced superoxide anions in the mitochondrial fraction [46]. As such, the potential for pharmacological modulators of mechanosensing to improve vascular function is of great clinical interest. Therapeutic strategies include targeting modifiers of transcription factors downstream of FSS transduction [47]. For example, lipid-lowering statins (HMG-CoA reductase inhibitors) in the systemic circulation that activate KLF_2_ (Kruppel Like Factor 2) [48]. KLF_2_ is regarded as a ‘master regulator of flow-induced gene expression in endothelial cells’, upstream of both eNOS and endothelin-1 activity [47].

Mimetics targeting specific mechanosensors are under development. The small-molecule agonist of mechanosensitive TRPV4 ion channels, GSK1016790A, has been shown to induce eNOS activation in coronary artery ECs [49]. Furthermore, oral administration of GSK1016790A reduced plaque formation in an atherosclerotic mouse model of [49]. Piezo1 channels can be activated with a specific synthetic compound, Yoda1. Responsiveness to Yoda1 has been demonstrated in the placenta, whereby HUVECs and microvascular FpECs exhibit increased intracellular Ca^2+^ entry [12]. Yoda1 also increases eNOS phosphorylation and blunts the effect of inflammatory cytokine, TNFα [50]. However, Yoda1 itself does not have the physico-chemical properties of a drug suitable for therapeutic use. New research shows that Piezo1 in HUVECs can be activated by shear stress induced by ultrasound stimulation (1 MHz for 10 s). These findings highlight the possibility of new interventions modulating specific FSS-sensing targets [51].

In pregnancy, efforts to enhance NO bioavailability have included maternal nitrite supplementation, although no clinically beneficial effects for FGR have yet been established [52]. A small study of transdermal nitroglycerin plus plasma expansion increased fetal weight in pregnancies affected by hypertensive FGR [53]. Increased maternal cardiac output and reduced total vascular resistance suggest that this effect was primarily due to alterations in maternal haemodynamics. In pregnancies with normal placental function, nitroglycerin reduced uterine vascular impedance, with no effect on fetal perfusion [54]. As such, any mechanistic effect of these treatments on fetoplacental vasculature remains to be determined.

Sildenafil citrate is under consideration as a rescue therapy for FGR. This vasodilating molecule increases NO concentrations by inhibiting phosphodiesterase-5 activity [55]. In an ovine FGR model, sildenafil increased both fetal and placental weights. This suggests that changes to growth are at least, in part, due to fetoplacental modifications, although umbilical artery resistance was not significantly affected by sildenafil [56]. Phosphodiesterase-5 mRNA and protein has been demonstrated in human chorionic arteries [57]. Here, sildenafil produced dose-dependent vasodilatation of chorionic arteries which was cGMP-dependent. Moreover, sildenafil-induced vasodilatation enhanced the vasodilation produced by the NO donor, SNP [57]. In a rabbit model of FGR, sildenafil was associated with increased numbers of dilated placental capillaries, venules, arterioles and arterial sinuses [55]. As such, sildenafil appears to have an effect on fetoplacental vasculature, and impact on fetal weight in animal models. However, high quality clinical studies of sildenafil have not yet improved pregnancy outcomes in severe early-onset FGR [58].

## 7 Conclusion

Mechanosensing by FpECs ultimately regulates NO bioavailability, thus impacting upon vasomotor tone. Mechanisms of FSS-sensing are compromised in FGR, allowing vasoconstrictor and anti-angiogenic effects to dominate. The fetoplacental endothelium in FGR attempts to compensate restricted blood flow by upregulating components of the NO system but this of course lacks flow responsiveness and is already maximal, which may explain why efforts to boost NO have not yielded clinically significant results. Critical to the success of a therapy for placental insufficiency will be a more nuanced understanding of how FSS is transduced by the fetoplacental endothelium, the interplay with stressors such as hypoxia, and ensuring that target vessels are responsive to NO-driven vasodilatation. We suggest that mechanosensors, including Piezo1, are an entry point to this new understanding and present an opportunity for targeted intervention. In addition, new computational models may be used to identify localised areas of fetoplacental circulatory deficiency, bridging the gap between better understanding fetoplacental haemodynamics and useful clinical interventions [38].

## Funding

Clinical Research Training Fellowship from the 10.13039/501100000265Medical Research Council, the 10.13039/501100000682Royal College of Obstetricians and Gynaecologists (MR/P002099/1 to LCM), Wellcome Trust Institutional Strategic Support Fund (to LCM) and BHF Programme (RG/17/11/33042) and Wellcome Trust Investigator Grants (110044/Z/15/Z) to DJB.
